# 3-Hydroxybutyrate as a Metabolite and a Signal Molecule Regulating Processes of Living Organisms

**DOI:** 10.3390/biom11030402

**Published:** 2021-03-09

**Authors:** Justyna Mierziak, Marta Burgberger, Wioleta Wojtasik

**Affiliations:** Faculty of Biotechnology, University of Wroclaw, 51-148 Wroclaw, Poland; justyna.mierziak@uwr.edu.pl (J.M.); marta.burgberger@uwr.edu.pl (M.B.)

**Keywords:** 3-hydroxybutyrate, polyhydroxybutyrate, 3-HB metabolism, signaling, animals, bacteria, plants

## Abstract

3-hydroxybutyrate (3-HB) as a very important metabolite occurs in animals, bacteria and plants. It is well known that in animals, 3-HB is formed as a product of the normal metabolism of fatty acid oxidation and can therefore be used as an energy source in the absence of sufficient blood glucose. In microorganisms, 3-HB mainly serves as a substrate for the synthesis of polyhydroxybutyrate, which is a reserve material. Recent studies show that in plants, 3-HB acts as a regulatory molecule that most likely influences the expression of genes involved in DNA methylation, thereby altering DNA methylation levels. Additionally, in animals, 3-HB is not only an intermediate metabolite, but also an important regulatory molecule that can influence gene expression, lipid metabolism, neuronal function, and overall metabolic rate. Some of these effects are the direct effects of 3-HB itself, while others are indirect effects, regulated by the metabolites into which 3-HB is converted. One of the most important regulatory functions of 3-HB is the inhibition of the activity of histone deacetylases and thus the epigenetic regulation of many genes. Due to the number of functions of this compound, it also shows promising therapeutic properties.

## 1. Introduction

3-hydroxybutyrate (3-HB) is not only a very important metabolite, but also a regulatory molecule that occurs in bacteria, animals, humans and, according to recent scientific reports, also in plants. This compound has a therapeutic effect on many human diseases, such as cancer or diseases of the nervous and circulatory systems. It also plays a useful role in veterinary diagnostics, where it is considered the most valuable diagnostic parameter among all ketone compounds. It is worthy to note that the homopolymer of 3-hydroxybutyrate, i.e., poly-3-hydroxybutyrate (PHB) is considered a good substitute for plastics, which is used e.g., in medicine, pharmacy, agriculture and in the food industry.

## 2. 3-Hydroxybutyrate Structure

3-hydroxybutyrate is an anion of a hydroxy acid that is the conjugate base of the 3-hydroxybutyrate acid, obtained by deprotonation of the carboxyl group. This compound in its structure contains a butyric acid core with a single hydroxyl substituent in position 3. 3-HB is a chiral molecule. There are two enantiomers of this compound, R/D, and S/L. R-3-HB is a metabolic product of mammals. Only R-3-HB is produced by normal metabolic processes. Hunger, exercise, the ketogenic diet, and any other condition causes endogenous production of only the enantiomer R-3-HB. Despite the fact that S-3-HB is not a normal product of mammalian metabolism, S-3-HB-CoA is an intermediate in the final stage of β-oxidation of fatty acids. The S-3-HB enantiomer can also bind to some cell receptors, but less strongly than R-3-HB [1]. In microorganisms, depending on the species, the R-3-HB or S-3-HB enantiomer can be synthesized [2]. In plants, there is currently no information on the presence of specific 3-HB enantiomers.

## 3. 3-Hydroxybutyrate Metabolism

In animals, the synthesis of beta-hydroxybutyrate takes place mainly in the mitochondria of the liver and begins with the condensation of two acetyl-CoA molecules to form acetoacetyl-CoA in a reaction catalyzed by beta-ketothiolase. Then, 3-hydroxy-3-methylglutaryl-CoA synthase (HMG synthase) catalyzes the condensation of acetoacetyl-CoA with an acetyl-CoA molecule to form 3-hydroxy-beta-methylglutaryl-CoA (HMG-CoA), which is degraded by HMG-CoA lyase to acetyl-CoA and acetoacetate, which is subsequently converted to 3-hydroxybutyrate by phosphatidylcholine-dependent mitochondrial beta-hydroxybutyrate dehydrogenase [3]. In animals, 3-HB is an intermediate metabolite of fatty acids. There are some indications from which it can be supposed that in addition to the main 3-HB pool derived from the synthesis in the liver, part of the 3-hydroxybutyrate pool in the animal’s body derives from the degradation of the polyhydroxybutyrate produced by the microbiome. This aspect is discussed in detail in the review by Bonartsev et al. [4].

In microorganisms, 3-HB can be synthesized through two pathways. One of these involves the direct synthesis of 3-HB by condensation of two acetyl-CoA molecules, which originate from the TCA cycle, to acetoacetyl-CoA by β-ketothiolase followed by reduction to 3-hydroxybutyryl-CoA by acetoacetyl-CoA reductase. The last step is the removal of the CoA group [5]. The second route is the synthesis of PHB followed by enzymatic depolymerization to 3-HB. The enzymatic depolymerization of PHB consists of at least two soluble factors, an activator and depolymerase. The synthesis of PHB also begins with the condensation of two acetyl-CoA molecules, by β-ketothiolase, followed by product reduction by acetoacetyl-CoA reductase. The last step in this metabolic pathway is the esterification of monomers to polyhydroxybutyrate. This reaction is catalyzed by PHB synthase. This enzyme catalyzes the stereoselective conversion of the 3-hydroxybutyryl-CoA precursor to polyoxoesters with the simultaneous release of CoA. An ester bond is formed between the carboxyl group of one monomer and the hydroxyl group of the adjacent monomer [6,7].

In bacteria, PHB is present in the form of intracellular cytoplasmic granules. PHB granules in vivo consist of an amorphous polymer core and a surface layer of structural and functional proteins. About 98% of the granules is PHB; the remainder consists mainly of proteins and lipids. In addition, PHB granules are associated with enzymes involved in the synthesis and degradation of PHB [8,9]. Intracellularly stored PHB may also be released into the environment due to bacterial cell death and lysis, and then degraded by extracellular PHB depolymerases to form 3HB [10]. Exogenous 3-hydroxybutyrate can be used by various soil bacteria as a source of carbon and energy. However, it is not known what transporter is responsible for the uptake of 3-hydroxybutyrate. In *Bacillus subtilis*, the HbuT transporter is presumed to perform this function [11].

**Figure 1:**
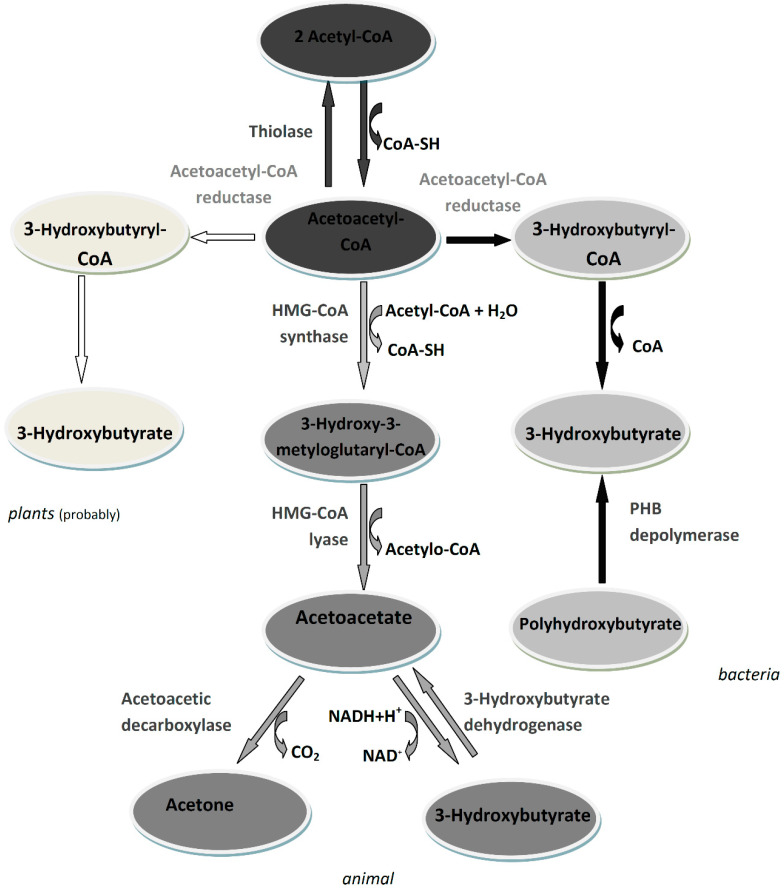
Metabolism of 3-hydroxybutyrate in living organisms.

The metabolism of 3-HB in plants is not fully understood and characterized. It is known that there is a substrate in plant cells and the active enzymes β-ketothiolase (EC 2.3.1.9) and acetoacetyl-CoA-reductase (EC 1.1.1.36), which are involved in the synthesis of 3-HB in bacterial cells [12,13,14,15]. On this basis, it can be assumed that 3-HB is also synthesized in plants. The metabolism of 3-hydroxybutyrate in living organisms is shown in Figure 1.

In animals, the transport of 3-HB is less known than its synthesis. As a small polar molecule, 3-HB easily dissolves in water and blood. The SLC16A6 monocarboxylate transporter may be a key transporter for 3-HB export from the liver, but transporters that allow for 3-HB uptake into target tissues or its intracellular movement remain unidentified. Several monocarboxylic acid transporters, including MCT1 and MCT2, carry 3-HB across the blood-brain barrier, and their expression can regulate uptake of 3-HB by the brain [16]. The renal elimination of ketone bodies by the kidney requires a balance of glomerular filtration and tubular reabsorption. The renal reabsorption capacity of 3-HB is limited by the maximum tubular transport capacity, suggesting that the reabsorption is mediated by specific transporters. The renal tubules reabsorb ketone bodies through two types of transporters: the sodium dependent monocarboxylate transporters (SMCT) in the luminal membrane and the monocarboxylate transporters (MCT) in the basolateral membrane. SMCTs use a sodium gradient generated by the basolateral Na-K-ATPase to transport monocarboxylates into the cell. SMCT transporters facilitate urate uptake by the loss of anion transporter 1 (URAT1), in the luminal membrane of the proximal tubule, which trap urate in exchange for an intracellular monovalent anion such as the 3-HB anion. It has been shown that MCT1-MCT4 transporters can transport ketone bodies across the plasma membrane in an H+-dependent and electro neutral manner. MCTs facilitate the basolateral exit of ketone bodies, which can be used for hepatic gluconeogenesis and as energy substrates in the brain and heart, respectively; ketone bodies are a preferred renal tubular energy source. The kidney tubules are highly reabsorbable and able to utilize ketone bodies. Kidney reabsorption and preservation of 3-HB during starvation may increase to fully cover the energy needs of the kidneys [17].

In target tissues, 3-HB is oxidized to acetoacetate (AcAc) in a reaction catalyzed by 3-HB dehydrogenase. Then, in the ketolysis step, succinyl-CoA-3-keto CoA transferase (SCOT) converts AcAc and succinyl-CoA into AcAc-CoA and succinate. AcAc-CoA is then cleaved by acetyl-CoA transferase to form two acetyl-CoA molecules that can enter the Krebs cycle. The metabolism of 3-HB does not require the use of ATP [18].

Ketogenesis in the human body occurs in response to energy requirements with insufficient carbohydrate intake. This condition is signaled by low insulin levels, high cortisol and glucagon. Insulin signaling leads to inactivation of the FOXA2 transcription factor by phosphorylation and nuclear export, while glucagon activates FOXA2 by acetylation. FOXA2 binds to the HMGCS2 promoter (3-hydroxy-3-methylglutaryl-CoA 2 synthase) and activates transcription. HMGCS2 transcription is also regulated by mTORC1 kinase (mammalian target of rapamycin kinase). This pathway is activated by insulin signaling or high levels of glucose or amino acids. The mTORC1 complex inhibits the peroxisome proliferator-activated alpha receptor (PPARa) and PPARa in turn is required for the induction of the growth factor FGF21 to activate ketogenesis. Hence, inhibiting mTOR may promote ketogenesis. HMGCS2 activity is also regulated post-translational by succinylation and acetylation [16]. Many enzymes involved in the metabolism of ketone bodies are susceptible to modification by SIRT3 and SIRT5 deacetylases. An example of such an enzyme is mitochondrial hydroxy-3-methylglutaryl-CoA synthase 2 deacetylated activated by SIRT3 [19,20]. There is also autoregulation of 3-HB production by inhibiting lipolysis by the PUMA-G nicotinic receptors in adipocytes and reducing the availability of fatty acids for ketogenesis. An important regulatory element of the level of 3-HB in the body is the activity of 3-HB dehydrogenase, which modulates the mitochondrial redox potential and ensures oxidation of ketone bodies in proportion to the needs of the ATP cell pool [21].

## 4. 3-Hydroxybutyrate as a Metabolite

3-hydroxybutyrate is the main representative of ketone bodies in animal cells, which are an alternative product of fatty acid oxidation. Therefore, 3-HB can be used as an energy source in the absence of sufficient glucose in the blood, which is of particular importance during starvation or illness. Another important function of ketone bodies is to provide acetoacetyl-CoA and acetyl-CoA for the synthesis of cholesterol, fatty acids, and complex lipids. In humans, 3-HB is well tolerated at low concentrations, but at high concentrations it can lead to ketoacidosis and death. Research indicates that a slight increase in the concentration of 3-HB may be important in some physiological states as it may modulate signaling cascades involved in cell growth, proliferation and defense against oxidative stress [22]. 3-HB is characterized by good penetration of cell membranes as well as fast penetration through peripheral tissues. For this reason, it shows good therapeutic properties during cellular stress caused, among other things, by extensive burns, hemorrhagic shock, hypoxia, and ischemia of organs and tissues [22,23].

In humans, the basal level of 3-HB in serum is in the low micromolar range but begins to rise to several hundred micromoles after 12–16 h of fasting, reaching 1–2 mM after 2 days without food and 6–8 mM after a long starvation diet. A 3-HB level of 1–2 mM can be achieved after 90 min of strenuous physical exercise. A level above 2 mM of 3-HB in the blood is also achieved by following a ketogenic diet that is almost carbohydrate-free. Babies produce and use 3-HB more efficiently than adults, as it is crucial immediately after birth when the brain uses ketone bodies for energy. In addition, in the early postpartum period, acetoacetate and 3-hydroxybutyrate are preferred over glucose as substrates for the synthesis of phospholipids and sphingolipids as required for brain growth and myelination. The level of 3-HB in children can reach 2–3 mM, similar to what is seen in the serum of elderly people after eating a ketogenic meal [24,25,26].

## 5. 3-Hydroxybutyrate as a Regulatory Molecule

**Figure 2:**
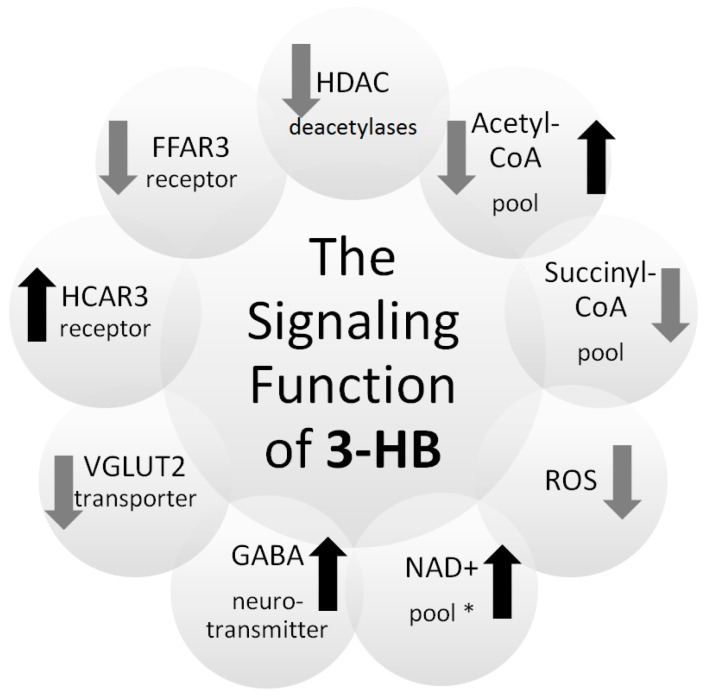
Regulatory functions of 3-hydroxybutyrate. 3-HB–3-hydroxybutyrate, CoA-coenzyme A; FFAR3–free fatty acid receptor 3; GABA–γ-amino butyric acid, HDAC–histone deacetylases, HCAR2–hydroxycarboxylic acid receptor 2; NAD–nicotinamide adenine dinucleotide; VGLUT–vesicular glutamate transporter. black arrow–activation, grey arrow–inhibition; * compared with glucose metabolism.

3-hydroxybutyrate also has important regulatory functions (Figure 2). It is a ligand for at least two cell surface receptors: hydroxycarboxylic acid receptor 2 (HCAR2) and the free fatty acid receptor 3 (FFAR3) [21,27]. The activation of the HCAR2 receptor by 3-HB reduces lipolysis in adipocytes, which may influence the regulation of the availability of precursors in the metabolism of ketone bodies [21,28]. HCAR2 is also activated in many other cell types, including immune cells, microglia and colonic epithelial cells, where its activation leads to an anti-inflammatory effect [29]. FFAR3 receptors are present in the sympathetic ganglia, and 3-hydroxybutyrate, by attaching to them, may affect the regulation of the overall metabolism of the body [21,27,28]. FFAR3 plays a role in the maintenance of glucose homeostasis in the body and influences the regulation of inflammation [30].

3-hydroxybutyrate appears to have a direct regulatory effect on the neuronal vesicular glutamate transporter (VGLUT2). Acetoacetate, 3-HB, and pyruvate inhibit Cl-dependent glutamate uptake, and the kinetics indicate competition for the Cl binding site. A biologically significant effect on glutamate uptake can occur at a physiologically low millimolar 3-HB level. Due to the inhibition of VGLUT2, 3-HB can reduce the excitatory neurotransmission of glutamate [31].

Although 3-HB is structurally similar to the neurotransmitter GABA (gamma-aminobutyric acid), there is no evidence for a direct effect of 3-HB on the activation of GABA receptors. However, metabolism of 3-HB can influence the production of GABA by increasing the synthesis of glutamate [32,33]. Additionally, research on isotopically labeled 3-HB shows that it can be used as a substrate for the synthesis of glutamine and other amino acids [32].

Another important aspect of the regulatory function of 3-hydroxybutyrate is its effect on the metabolism of reactive oxygen species and the maintenance of cellular redox homeostasis. 3-hydroxybutyrate can act as a direct antioxidant for hydroxyl radical. What is more, it can inhibit mitochondrial ROS production as well as promote transcriptional activity of antioxidant defenses [34].

Literature data show that one of the most important properties of 3-HB is being an inhibitor of histone deacetylases (HDAC). Histone deacetylases are a protein family that plays an important role in regulating gene expression. Histone hyperacetylation is usually associated with the activation of gene expression, and HDAC activity reduces the level of gene expression. Additionally, non-histone proteins can be deacetylated by HDAC, including NF-κB, TP53, p53, c-Myc, MyoD [35,36,37,38]. The inhibition of HDAC class I appear to be a direct effect of 3-HB action. Enzymatic inhibition of HDAC by 3-HB is observed in in vitro assays using purified, labeled HDAC and synthetic peptide substrates. Competitive inhibition is the likely mechanism for HDAC inhibition by 3-HB. The carboxyl group of the inhibitor usually binds competitively to catalytic zinc at the bottom of the hydrophobic channel of the active site of HDAC [39,40]. 3-HB can also promote the protein hyperacetylation indirectly. The catabolism of 3-HB to acetyl-CoA can raise intracellular levels of acetyl-CoA, providing an additional substrate for protein acetylation, thereby driving the equilibrium reaction towards acetylation. Summing up, 3-hydroxybutyrate may increase the level of acetylation of proteins, including histones, which translates into changes in the chromatin structure and gene expression [1,35]. It was observed that during the starvation of mice, an increase of 3-HB level in plasma was associated with an increase in histone acetylation in many tissues. Research by Shimazu et al. showed that the HDAC inhibition by 3-HB correlates with global changes in transcription, including genes encoding transcription factors involved in resistance to oxidative stress: FOXO3A and MT2 (metallothionein 2). The induction of FOXO3A appears to be a direct inhibitory effect of HDAC. Treatment with 3-HB increased the acetylation of histones in the promoters of these genes and led to a change in their expression [41].

However, it is worth noting that there are reports in the available literature which do not confirm that 3-HB is an inhibitor of HDAC, thus it does not increase the level of histone acetylation in animal cells [42,43].

3-HB can itself modify proteins at the post-translational level. β-hydroxybutyrylation of lysine was detected by mass spectrometry as a histone modification in yeast, fly, mouse and human cells. Western blot analysis showed that the treatment of human cells by 3-HB standard was associated with an increase in the level of hydroxybutyrylated histones. At least 40 lysine residue sites in histones have been detected in human cells (including sites critical to transcription regulation such as H3K9), which can be modified by 3-HB. Chromatin immunoprecipitation with specific antibodies also revealed that the β-hydroxybutyrylation of lysine 9 of histone 3 is related to promoters of actively transcribed genes. The exact mechanisms and their regulation involved in the addition and removal of hydroxybutyrylated lysine residues are not known, but it appears that such histone modification may play an important role in regulating gene expression during starvation and other processes associated with the increase in 3-HB concentration in the body [44].

Utilization of 3-HB in peripheral tissues consumes succinyl-CoA. This consumption of succinyl-CoA may affect the balance of lysine succinylation, which, as with acetylation, is widespread in the mitochondria and occurs in various organisms. A significant proportion of these succinylation sites are regulated by sirtuin 5 (SIRT5). The enzymatic activity of HMGCS2 in the liver is suppressed by succinylation and restored by SIRT5-mediated desuccinylation. The mechanism of lysine succinylation is not fully understood; it is assumed that this is a non-enzymatic process depending on local succinyl-CoA concentrations. Numerous enzymes involved in fatty acid oxidation, amino acid catabolism and the TCA cycle are strongly succinylated and regulated by SIRT5. Utilization of succinyl-CoA during 3-HB metabolism may reduce the level of succinylation of these proteins and indirectly regulate the pathways in which they are involved. During the metabolism of 3-HB, fewer NAD+ molecules are used than during the metabolism of glucose. The cellular compartment is different in both cases. 3-HB metabolism takes place in the mitochondria, preserving the cytoplasmic NAD pool. Preservation of the pool of cytoplasmic NAD+, a cofactor of many important enzymes, may have important cellular effects during the metabolism of 3-HB [1].

## 6. Therapeutic Properties of Beta-Hydroxybutyrate

3-hydroxybutyrate, as an important metabolite and regulatory molecule, has many therapeutic properties in many human diseases.

### 6.1. Cardiovascular Diseases

3-hydroxybutyrate has a beneficial effect against hypertension, which is a chronic disorder that is often associated with cardiovascular and renal complications [45]. It has been shown that supplementation with β-hydroxybutyrate precursor 1,3-butanediol has a positive impact on reducing arterial hypertension to the same extent as exercise due to the inhibition of the renal inflammasome Nlrp3 by 3-hydroxybutyrate [46,47]. Furthermore, 3-HB exhibits a protective effect against myocardial ischemia–reperfusion injury. The animal studies shown that the prophylactic use of 3-hydroxybutyrate not only reduced the area of ischemic necrosis (infarction), but also significantly reduced apoptosis caused by injury. What is more, 3-HB reduced levels of cardiac troponin I, creatine kinase and lactate dehydrogenase in serum. In addition, the use of 3-hydroxybutyrate stimulated the autophagic flux and decreased the amount of mitochondrial reactive oxygen species formed, increased ATP production, and decreased mitochondrial swelling and partially maintained the potential of mitochondrial membranes in the heart muscle [48,49]. It is also noteworthy that the increase of the amount of 3-hydroxybutyrate in blood is a potential new treatment for heart failure with reduced ejection fraction [49].

### 6.2. Cancer

Ketone bodies, including 3-hydroxybutyrate, play a metabolic and epigenetic role in neoplasms, but their action may both counteract and promote tumor progression. 3-hydroxybutyrate owes its anti-cancer properties mainly to the fact that it is an HDAC inhibitor. It selectively activates genes encoding cell cycle blocking proteins, such as the protein p21WAF1, which leads to cell apoptosis. The mechanism of action is based on blocking cdk2 kinase by stimulating the expression of p21WAF1. Additionally, expression of cyclins is inhibited, which stops cells in the G1 growth phase and blocks cell division. Increased histone acetylation is also noticeable in normal cells, but the anti-proliferative and pro-apoptotic effects are enhanced in cancer cells [50,51]. The anti-tumor effect of 3-HB occurs through the inhibition of histone deacetylases, while the pro-tumor effect manifests itself in the faster development of the tumor. Such different behavior was observed in the example of butyrate, which was effective against some types of cancer and promoted others. This “butyrate paradox” is explained by the effect of butyrate on the acetylation of histones, and thus the inhibition of the proliferation of cancer cells, which are focused on the use of glucose (Warburg effect), while in cells that oxidize butyrate as an energy source, it does not reach a sufficient level to inhibit tumor growth. A study by Rodrigues et al. confirmed the existence of an analogous paradox in the case of 3-hydroxybutyrate alone [52]. It is presumed that the action of 3-HB and other ketone bodies may have both therapeutic and protective effect against various cancers including breast cancer, brain tumors, colon cancer, as well as lung cancer [53,54,55,56,57].

### 6.3. Nervous System Diseases

3-hydroxybutyrate may also play an important role in the prevention and treatment of neurodegenerative diseases [58,59]. The accumulation of β-amyloid peptide (Aβ), inflammation and oxidative stress contribute to the complex pathogenesis of Alzheimer’s disease. In animal model studies, the use of 3-hydroxybutyric acid was found to improve cognitive function and to reduce Aβ peptide accumulation and microglia overactivation in the brain. 3-hydroxybutyric acid also enhances mitochondrial respiratory function of hippocampal neurons and protects them from the toxic effects of Aβ peptide. The enzymes APP (β-amyloid precursor protein) and NEP (neprilysin) are regulated by this acid through the G109A protein-coupled receptor (GPR109A) [60]. One of the neuroprotective mechanisms of β-hydroxybutyrate and acetoacetate to protect against Alzheimer’s disease is based on blocking the amyloid-β 42 entry into neurons. Prevention of intracellular amyloid-β 42 accumulation protects against mitochondrial complex I inhibition, reduces oxidative stress, and improves neuronal plasticity [61].

Parkinson’s disease is the second most common neurodegenerative disorder, whose main characteristic is the loss of dopaminergic neurons in the substantia nigra par compacta (SNpc). It is believed that excessive activation of microglia and the resulting release of pro-inflammatory cytokines (TNF-α, IL-1β, IL-6) or pro-inflammatory enzymes (iNOS, inducible nitric oxide synthase; COX-2, cyclooxygenase-2) contribute to neurodegenerative processes; therefore, inhibition of excessive microglia activation may be a potential therapeutic strategy to prevent further progression of this disease. 3-hydroxybutyric acid treatment significantly reduces motor dysfunction in a rat model of Parkinson’s Disease. This effect was due to inhibition of microglia overactivity, via the G109A protein-coupled receptor (GPR109A), and involved the NF-κB signaling pathway, resulting in inhibition of the production of proinflammatory enzymes (iNOS and COX-2) and proinflammatory cytokines (TNF-α, IL-1β, and IL-6) and protection of dopaminergic neurons in the midbrain black matter [62,63].

Huntington’s disease is a genetic disorder of the nervous system that is caused by the loss of function of the protein huntingtin, caused by too many repeats of the glutamine-encoding trinucleotide. Abnormalities in mitochondrial function and epigenetic regulation are believed to be critical in this disease. Based on the research findings, it is suggested that by simultaneously targeting mitochondria and epigenetic abnormalities, D-3-hydroxybutyrate may be a valuable therapeutic agent in Huntington’s disease, as in a mouse model of Huntington’s disease; it was observed that 3-HB infusion reduced motor deficits and microgliosis, and prolonged lifespan and prevented histone deacetylation in the striatum. In PC12 cells with induced expression of mutant huntingtin, D-3-hydroxybutyrate was found to prevent histone deacetylation through a mechanism independent of its mitochondrial action and independent of inhibition of histone deacetylases. It has also been shown that usage of a ketogenic diet, which involves increasing ketone bodies in a mouse model of Huntington’s disease (R6/2 1J), delays weight loss which is a hallmark of the disease [64,65].

Inflammation of the nervous system is strongly associated with the pathophysiology of depression. Studies suggest that a small amount of 3-HB produces an antidepressant effect, possibly through anti-inflammatory mechanisms, and that the prefrontal cortex is a region involved in the antidepressant effects of 3-HB [66]. Moreover, 3-HB may be a therapeutic candidate for the treatment of stress-related mood disorders as it exerts antidepressant-like effects, possibly by inhibiting NLRP3 inflammasome-induced neuro-inflammation in the hippocampus (a macromolecular protein complex that is part of the body’s immune response; its activation enables the secretion of proinflammatory cytokines, but its excessive activation may contribute to the development of pathophysiological processes) [67]. Another study showed that 3-HB, by inhibiting the NLRP3 inflammasome activation in glioblastoma cells, consequently inhibits the migration of cancer cells [68].

### 6.4. Diabetes

In diabetics in the absence or deficiency of insulin with elevated levels of counter-regulatory hormones, there is a high blood glucose concentration with a simultaneous deficit in the cells. Cells must obtain energy from another source, which may be the breakdown of fatty acids with the simultaneous formation of ketone bodies as by-products. In diabetics, beta-hydroxybutyrate can therefore be used as an energy source for organs such as the heart, partially replacing glucose [69]. The ketogenic diet can help with glycemic control in people with diabetes. Cardiovascular disease is the leading cause of death among adults with type 2 diabetes. Research by Bhanpuri et al. showed that following a ketogenic diet for one year had a significant impact on reducing risk factors for cardiovascular disease [70]. Ketogenic diets have yielded positive results, to the point of reversing type 2 diabetes in 54% of patients in one 2-year study, however, it is unclear if the benefit arose from a ketone specific mechanism. It is a fact that, ketogenic intervention lowers blood glucose levels and improves insulin secretion and improves the lipid profile of diabetic patients [59]. However, when the process of deriving energy from fatty acids is carried out very intensively (e.g., in patients with poorly controlled diabetes), there is an accumulation of ketone bodies in the organism, which can lead to the dangerous diabetic ketoacidosis (DKA). The high concentration of ketone bodies, due to their acidic pH, affects the electrolyte balance and disturbs life processes, causes damage and dehydration of cells, as the organism strives to eliminate their excess in the urine. Diabetic ketoacidosis is characterized by a serum glucose level greater than 250 mg per dL, a pH less than 7.3, a serum bicarbonate level less than 18 mq per L, an elevated serum ketone level, and dehydration [71,72,73]. It has been shown that the development of ketoacidosis leads to inflammation, characterized by increased levels of pro-inflammatory cytokines and increased markers of oxidative stress which can lead to cellular damage of lipids, membranes, proteins, and DNA [74]. In addition, DKA can lead to hypokalemia, cerebral edema, pulmonary edema, and damage to the kidneys or other organs due to fluid loss. Less common problems can include rhabdomyolysis, thrombosis and stroke, pneumomediastinum, prolonged corrected QT interval, and memory loss with decreased cognitive function. DKA also negatively affects the circulatory system. In extreme cases, DKA may be fatal [72,75].

### 6.5. Ketogenic Diet

**Figure 3:**
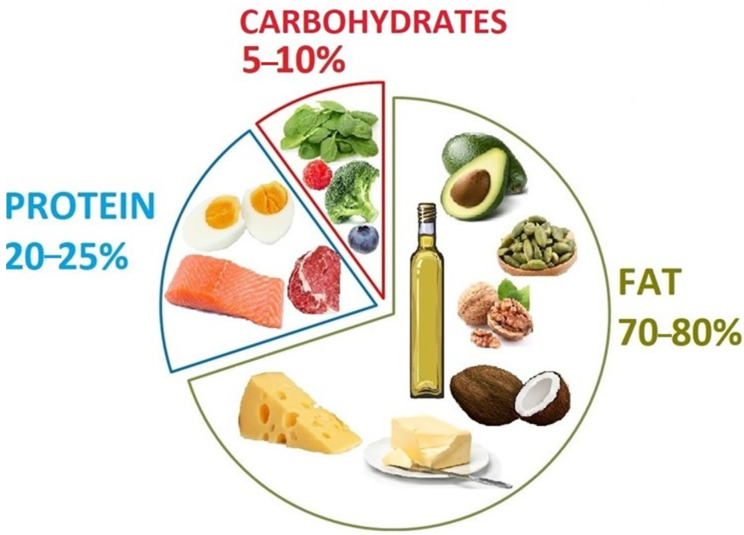
Distribution of macronutrients in a ketogenic diet.

In medicine, 3-hydroxybutyrate plays an important role in the therapeutic ketogenic diet (KD). The ketogenic diet is a high-fat, low-carbohydrate, low-protein (Figure 3) diet developed in the 1920s. This diet is based on increasing the concentration of ketone bodies, mainly beta-hydroxybutyrate, in the body and is mainly used in the treatment of drug-resistant epilepsy [76,77,78,79,80]. People following this diet showed a reduced number of seizures and the seizures were less severe. Nowadays, there has been renewed interest in the KD, especially in the treatment of drug-resistant epilepsy in children, but also in other medical conditions and weight reduction [77,79]. The mechanisms of KD to reduce epileptic seizures are not fully known, but it has been suggested that such properties may be influenced by alteration of amino acid distribution in the brain favoring increased synthesis of the inhibitory neurotransmitter gamma aminobutyric acid and the effect of glucose restriction and the effect of altered fatty acid metabolism [76,78,80].

The ketogenic diet has a beneficial effect in counteracting cancer progression due to the low content of carbohydrates, which results in a reduced availability of an energy source for the tumor (reduced blood glucose level), and thus its abnormal metabolism. Studies conducted on many animal models available preclinical and clinical evidence indicate that KD may reduce the size of the tumor and increases the average survival time and increase in quality of life [81,82] as well as may be helpful in reducing the negative effects of neoplastic cachexia [83]. The ketogenic diet in the treatment of cancer was described in detail in the review Weber et al. [84].

Following a ketogenic diet may be beneficial in the treatment of many other diseases and medical conditions, such as polycystic ovary syndrome, metabolic disease, ischemia of heart and brain injury [85,86]. In addition, the use of a ketogenic diet in overweight or obese people allows a rapid and sensible weight loss with favorite biomarker changes and improvement in glycemic control [87,88].

However, it should be remembered that KD is a high-fat diet and low in carbohydrates, which may also have adverse effects, such as increases in LDL-C and very-low-density lipoproteins (VLDL), muscle cramps, bad breath, changes in bowel habits, loss of energy, hepatic steatosis, hypoproteinemia, reduced workout efficiency, and vitamin and mineral deficiencies [87,89,90]. Many concerns about the use of KD are related to its effects on the circulatory system. Recent data indicate that a long-term use of the ketogenic diet can lead to cardiac fibrosis. In rats, KD decreased mitochondrial biogenesis, reduced cell respiration, and increased cardiomyocyte apoptosis and cardiac fibrosis. 3-HB acting as an HDAC2 inhibitor, promoted histone acetylation of the Sirt7 promoter and activated Sirt7 transcription, which in turn inhibited the transcription of mitochondrial ribosome-encoding genes and mitochondrial biogenesis, leading to apoptosis and cardiac fibrosis [91]. There is also other evidence of an adverse effect of 3-HB on the cardiovascular system. High levels of 3-HB have been observed in cardiac tissues in patients with atrial fibrillation, and increased levels of 3-HB have been associated with major adverse cardiovascular events in patients undergoing hemodialysis [91,92]. Cardiovascular diseases of unknown etiology have also been reported in clinical trials with KD users [93,94]. A high-fat diet can be dangerous not only for the heart but also for the brain. There are reports of cognitive decline due to this diet [95]. In addition, high fat consumption with a simultaneous limitation of carbohydrate consumption, which are a source of dietary fiber for microbiota, affects the unfavorable changes in the intestinal microflora and metabolomic profiles of feces [96]. High fat intake of KD-fed mice led to induce hepatic inflammation and development of nonalcoholic fatty liver disease [73,97]. There are also reports in the literature on the development of nephrolithiasis in people (mainly children) following a ketogenic diet [98,99,100]. KD is also associated with impaired bone health [101,102]. In conclusion, it can be stated that the ketogenic diet, apart from many positive properties, may also be associated with a number of risks. Particular care should be taken with the long-term use of this diet, and people with chronic diseases should consult a physician before introducing this diet.

## 7. The Importance of the Beta-Hydroxybutyrate Polymer for Living Organisms

3-hydroxybutyrate is a poly-3-hydroxybutyrate (PHB) monomer. PHB is a homopolymer that is a backup material for prokaryotes, being an important source of carbon and energy. It is produced by bacteria of the genera *Azotobacter*, *Bacillus*, *Cupriavidus*, *Pseudomonas*, *Ralstonia*, and *Rhizobium*, among others [103]. Its accumulation is especially important in times of starvation and during the action of stress factors as it increases the chances of survival of the microorganism. PHB synthesis is mainly stimulated by an excess of carbon source in the environment, with a simultaneous low availability of phosphorus and nitrogen or oxygen. PHB is stored as granules in inclusion bodies. The surface of the granule is covered with a layer of phospholipids and proteins. PHB is a short-chain polyhydroxyalkanoate (PHA) and belongs to the group of aliphatic polyesters [7]. PHB was first discovered by the French biologist M. Lemoigne in 1926 in the bacterium *Bacillus megaterium* [104].

PHB biosynthesis in bacteria involves the β-ketothiolase catalyzed condensation of two acetyl-CoA molecules that are derived from the TCA cycle, followed by reduction to 3-hydroxybutyryl-CoA and esterification of the monomers. During normal bacterial growth, β-ketothiolase is inhibited by free coenzyme-A derived from the Krebs cycle. During nutrient deficiency, the entry of acetyl-CoA into the Krebs cycle is limited and is directed to the PHB biosynthetic pathway. The most common carbon source for bacterial synthesis of PHB is sugars such as glucose and fructose [105].

PHB, due to the fact that it is insoluble in water, thermoplastic, enantiomerically pure, non-toxic, biocompatible, and especially biodegradable, is of great interest within academia and industry [106,107]. PHB is recognized as a good alternative substitute for plastics and has found tremendous applications in medicine, pharmacy, agriculture, food, automotive and painting industries. It is used in the production of automotive parts, sanitary equipment, electrical appliances, packaging and containers [108,109,110]. In vitro biocompatibility of PHA has been demonstrated in various cell cultures of fibroblasts, mesenchymal stem cells, osteoblasts, bone marrow cells, articular cartilage, chondrocytes, endothelial cells, and smooth muscle cells. The presence of PHB in cell cultures did not affect cell growth and migration, and this polymer turned out to be a biocompatible and biologically inert material. The compatibility with body tissues means that PHB can be used in medical areas such as the production of surgical sutures, dressings, implants, orthopedic pins, stents, medical devices and drug carriers. This polymer is used as an auxiliary material for cell growth in tissue engineering [111,112,113,114,115]. Many bacterial strains have the ability to synthesize PHB, but on an industrial scale, *Ralstonia eutropha* is of greatest importance. Most often, genetic engineering techniques are used to optimize and increase the production of biopolymers by microorganisms [5,109,112].

The availability of bacterial gene sequences needed for PHB biosynthesis and the availability of advanced methods of molecular biotechnology in plants made it possible to produce this polymer in transgenic plants [116,117]. The expression of genes involved in the PHB synthesis in the cytosol of plants can hinder the survival and productivity of plants. This may be partly due to the loss of the cytosolic pool of acetyl-CoA. This effect may be reduced when PhaABC genes are expressed in plastids. For this purpose, appropriate genetic vectors or plastid targeting sequences in the gene construct are used. The expression in plastids also allows higher levels of PHB to be achieved [117,118,119,120,121,122]. The highest PHB content was obtained in *Arabidopsis thaliana* and was equal to 4% of fresh weight [119]. As in bacteria, polymer chains accumulate in plant cells in the form of granules [117]. The accumulation of PHB in plant tissues above a certain level causes undesirable changes in plant development and metabolism and leads to reduced biomass levels, chlorophyll deficiency and reduced fertility. While the reasons for this are unknown, several structural and metabolic factors need to be considered. PHB is an osmotically and metabolically inert polymer, but its presence in the form of a hydrophobic granule can be problematic. In prokaryotes, PHB granules are coated with P proteins, the so-called phasin proteins, which provide a barrier between the hydrophobic granule and the bacterial cytoplasm, and also prevent non-specific binding of other proteins [123,124]. Many studies have shown that these proteins can bind polyhydroxyalkanoate granules in vivo and in vitro in various bacterial strains that do not naturally produce these polymers [125]. The co-expression of genes encoding enzymes of PHB synthesis and proteins, the so-called phasin proteins, did not improve the plant phenotype [126]. It has been observed that in plants producing PHB in chloroplasts, the granules of this polymer colocalize with plastoglobulins, which leads to the reduction of plastoglobulins and disrupts the photosynthesis process [120,127]. Many metabolic changes were observed in plants producing PHB, including differences in the levels of organic acids, amino acids, sugars and sugar alcohols, lower levels of starch and sucrose and chlorophyll [117,119,128]. Due to the fact that PHB synthesis uses reducing equivalents the NADPH/NADH ratio in the plant changes. This change can alter the redox balance and the activity of many enzymes, including those involved in the Calvin cycle, photosynthesis, and fatty acid biosynthesis. These processes are also regulated by the availability of a substrate that can be shifted to the synthesis of PHB [117].

Polyhydroxybutyrate is synthesized mainly by some prokaryotic organisms. It is known that in all organisms (bacteria, yeast, plants, animals) the synthesis of short-chain PHAs with up to 150 units is possible. However, these compounds are present in a very low concentration in cells. In eukaryotes, they usually bind covalently to various proteins. They most likely take part in the functioning of calcium channels and DNA transport and have a protective function of proteins to which they bind [129]. Tsuda et al. in 2012 for the first time discovered that polyhydroxybutyrate can be produced in the rice root under the influence of externally provided acetic acid [130]. The team also carried out studies on other plants that had different types of photosynthesis, such as wheat, radish (C3 photosynthesis), maize (C4 photosynthesis), and *Kalanchoe pinnata* (CAM photosynthesis) [15,130]. These studies showed that the synthesis of PHB in higher plants is possible regardless of the type of photosynthesis performed. Tsuda et al., based on the results of their research, believe that PHB is synthesized naturally in plants. In addition, acetic acid provided externally to rice seedlings led to an increased PHB content. The authors suggest that treating plants with acetic acid leads to degradation of PHB, and the supplied acetic acid is incorporated into the newly formed PHB chains. These data may prove that PHB in plants is not only a carbon reserve. Of the three enzymes involved in the synthesis of PHB in bacteria, two homologues are found in plants: beta-ketothiolase and acetoacetyl-CoA reductase [12,13]. Tsuda et al. in their study on rice seedlings revealed enzymatic activity localized in plastids similar to that of PHB synthase. The authors concluded that the existing activity of the three enzymes participating in PHB synthesis confirms the synthesis of PHB from acetyl-CoA in plants by a mechanism found in bacteria. However, in order to finally confirm these conclusions in further studies, it is necessary to purify the above enzymes and study the genes encoding them [15].

## 8. The Role of Beta-Hydroxybutyrate in Animals

The main function of 3-HB in animals is to provide energy. During difficult climatic conditions and difficult access to food, many animals, including domestic yak, use stored lipids as a source of energy. The main compound that is produced during their degradation is 3-hydroxybutyrate. The purpose of 3-hydroxybutyrate is not only to provide the animals with energy, but also as a signaling molecule to regulate lipid metabolism. Hunger causes weight loss and influences the activation of the cAMP/PKA/CREB signaling pathway (cyclic adenosine monophosphate (cAMP)/protein kinase A (PKA)/transcription factor (CREB) to promote lipid metabolism and maintenance of a low and stable glucose concentration. A study by Zou et al. on three yak groups (a control food group, a starving group and a starving group with beta-hydroxybutyrate infusion) showed that hunger enhances lipid catabolism, and the 3-HB infusion affects the regulation of gene expression. The increasing concentration of 3-HB can increase GPR109A mRNA expression in subcutaneous adipose tissue and inhibit the cAMP/PKA/CREB signaling pathway and inhibit lipid degradation. The level of 3-HB thus controls the utilization of lipid stores [131].

Other studies also confirm that 3-HB is not only a metabolic energy intermediate, but also has a signaling function in regulating energy expenditure and homeostasis during malnutrition in animals. 3-HB inhibits adipocyte lipolysis in mice and cattle. This compound can bind specifically e.g., with the receptor from GPCRs HM74a/PUMA-G expressed in adipocytes and regulates its own production, thus preventing ketoacidosis and promoting efficient use of fat stores [21,132]. The regulation of the sympathetic nervous system in animals plays an important role in balancing energy intake during excess or no food. Some metabolites can directly regulate the activity of this system via GPR41 (G-protein-coupled receptor 41, also called free fatty acid receptor 3 (FFAR3)), at the level of the sympathetic ganglion. 3-hydroxybutyrate, during starvation, can inhibit the activity of the sympathetic system by antagonizing GPR41, thereby controlling the body’s energy expenditure and helping to maintain metabolic homeostasis [27]. Too high concentration of 3-HB in the organisms of animals leads to many negative effects. In dairy cows, hyperketonemia has been associated with deterioration of the parameters of milk production and deterioration of the health condition of cows, poorer functioning of the immune system, negative effects on reproductive performance, an increased risk of certain diseases, such as gastrointestinal disorders, liver damage, inflammation of the uterus and udder, or lameness [133,134,135,136,137,138].

As the measurement of 3-HB in serum becomes more available, it has become useful in veterinary diagnostics. Measurement of 3-hydroxybutyrate and acetoacetate is used in the diagnosis of diabetic ketosis in animals, but the studies conducted by Gorman et al. indicate that it may also be a parameter taken into account in other diseases, as an increased level of it was observed in cats with chronic kidney disease, overactive thyroid gland or fatty liver. In addition, since ketones reflect the use of fat as an energy source in the absence of sufficient carbohydrate supply, the measurement of serum 3-HB could potentially be used as an indicator of caloric stress and the need for nutritional support in veterinary patients [139].

Hyperketonemia is one of the most common and costly metabolic disorders in highly productive dairy cows and its diagnosis is based on determination of the concentration of 3-hydroxybutyrate in the blood or milk. Among all ketone compounds, 3-HB is considered the most valuable diagnostic parameter because it is the most stable. Hyperketonemia is associated with higher fat content and lower levels of protein and urea nitrogen in milk. Ketosis worsens the health of dairy cows, and the economic losses are mainly due to impaired reproductive performance and a decrease in milk production efficiency. Therefore, early detection of hyperketonemia in dairy cows is essential [133].

3-hydroxybutyrate may also be an important marker of disturbances in dairy cows leading to liver damage. Research by Song et al. revealed that the ketotic dairy cows exhibited oxidative stress and liver damage. High concentrations of 3-HB increased the oxidative stress of bovine hepatocytes in in vitro tests, increased the expression of pro-apoptotic genes and inhibited the expression of anti-apoptotic genes and promoted apoptosis in bovine hepatocytes. N-acetyl-1-cysteine, glucose and SB203580 (a p38 inhibitor) significantly attenuated the apoptotic damage to hepatocytes induced by 3-HB. Based on the results of the research, it was concluded that 3-HB induces apoptosis of bovine hepatocytes through the ROS-p38-p53/Nrf2 signaling pathway [136]. The cytochrome P4502E1 (CYP2E1) plays an important role in inducing oxidative stress. It has been shown that high levels of 3-HB in cow hepatocytes significantly increase the expression and activity of hepatic CYP2E1 and may have an influence on the induction of oxidative stress in cows with clinical ketosis [140]. A high level of 3-HB also activates the NF-κB signaling pathway and increases the release of pro-inflammatory factors. High levels of 3-HB have been shown to induce inflammatory responses in bovine endometrial cells by activating NF-κB signaling mediated by oxidative stress [141]. In some cases, 3-HB may exhibit antioxidant properties. Many studies show that when the concentration of 3-HB is not too high, it shows an antioxidant effect and inhibits lipoperoxidation. Hydroxyl radicals can be efficiently removed by 3-HB. This compound also prevents the hypoglycemia-induced increase in lipid peroxidation in the rat hippocampus. Furthermore, in mammals, the metabolism of ketone bodies causes a more negative redox potential of the NADP antioxidant system, which is a terminal destructor of ROS. Ketone bodies are also able to inhibit mitochondrial ROS production. 3-HB is an inhibitor of histone deacetylases which influence the transcription of the FOXO3a gene; this results in the transcription of enzymes of the antioxidant pathways, which in turn leads to a significant reduction in markers of oxidative stress. In summary, 3-HB can increase the antioxidant capacity of the body through several mechanisms, and its level is important here [41,142,143,144,145,146,147]. In the case of bacteria, it was shown that methyl-esterified dimers and trimers of 3-hydroxybutyrate, produced by bacteria capable of polyhydroxybutyrate biosynthesis have 3 times more hydroxyl radical scavenging activity than glutathione and 11 times more activity than vitamin C or 3-HB monomer alone. In addition, the genes encoding the enzymes that degrade polyhydroxybutyrate are activated, resulting in the degradation of PHB and the production of ME-3HB oligomers in bacteria infecting plant cells and exposed to hydroxyl radical stress [148].

Determination of the level of 3-HB in the serum and in the fetal fluid is important, among other things, while monitoring pregnancy in animals, e.g., dogs. During pregnancy, there are many metabolic adaptation processes, including increased hepatic glucose secretion, decreased peripheral insulin sensitivity, and a lower level of peripheral insulin. These processes are designed to provide nutrients to the fetus while preserving euglycemia; however, glucagon and norepinephrine are inhibited during pregnancy, making it difficult for the body to overcome hypoglycemia. If the nutrient requirements of the fetus are too high, e.g., with a large litter, or the maternal nutrient supply is insufficient, a dangerous ketosis with severe hypoglycemia may develop, which may result in weakness, ataxia, collapse, convulsions, and even coma. Therefore, the monitoring the 3-HB level may provide an important marker of this condition and will allow the animal to receive appropriate veterinary care [149].

A high concentration of 3-HB may affect the reproduction of ruminants. When animals receive low-quality feed, their bodies experience energy deficits and an increase in the concentration of 3-HB, which in this case has two functions: it provides the necessary energy, but also acts as a signal molecule for adaptive changes in metabolic pathways. The increasing concentration of 3-HB in the serum of ruminants is an indicator of the metabolic state and their reproductive capacity. The coordination of metabolic and reproductive disorders occurs in the pituitary and hypothalamus. Research on gene expression in these tissues in sheep under the influence of 3-HB revealed altered gene expression in pathways related to stimulus perception, inflammation and cell cycle control. In the serum of the tested animals, an altered level of the components of the amino acid and pyrimidine metabolism was observed [150]. Ketonemia is relatively often observed in sheep in the last weeks of pregnancy as a result of an increased energy requirement of the developing fetus and insufficient supply of nutrients to the mother. A high level of 3-HB indicates malnutrition, which has negative health effects on ewes and lambs. Research by Ratanapob et al. showed that increased levels of 3-HB in sheep in late pregnancy were associated with a low body condition score, parasitic infection and an increased number of fetuses. Therefore, herd managers should monitor the blood level of 3-HB in sheep, especially if they exhibit any of the above risk factors [151].

Dimers and trimers of 3-hydroxybutyrate also play a role in the reproduction of spiders. They are synthesized de novo or with the use of microorganisms and act as spider sex pheromones. These pheromones are found on the female’s web and play a role in attracting males to her. These compounds also cause changes of male behavior, e.g., in *Linyphia triangularis*, which involves web reduction behavior of males on the webs of unmated adult females. The male thus reduces the likelihood of another male reaching the web and confronting it for the right to mate with the female. After mating, the female rebuilds her web without the pheromone [152,153].

## 9. The Role of Beta-Hydroxybutyrate in Plants

Little is known about the physiological importance of 3-HB in plants. Plant literature data focus mainly on the 3-HB polymer polyhydroxybutyrate, a product of plant transformation with bacterial genes, for its production for the biodegradable plastics industry. However, there are reports that PHB can also be synthesized naturally in plants [15,154]. The work of Mierziak et al. proves that 3-hydroxybutyrate occurs naturally in flax [155]. It is not yet possible to identify the origin of 3-HB in plants. As in animals, some of this metabolite in plant cells may be derived from symbiotic bacteria that produce PHB, which then biodegrades to 3-HB. The source of depolymerizable PHB may also be that synthesized naturally in plants. Another possibility is de novo synthesis from acetyl-CoA or another as yet undiscovered metabolic pathway. As mentioned earlier, plants contain enzymes that are involved in the synthesis of 3-HB in bacteria [15,130,155]. Flax with the introduced bacterial β-ketothiolase gene (the first enzyme in the 3-HB synthesis pathway) showed an increased content of 3-HB as well as overexpression of the endogenous plant beta-ketothiolase gene. This may suggest the metabolic control of gene expression in its synthesis pathway by 3-HB [155].

Research by Mierziak et al. indicates that, similarly to animal cells, 3-HB may act as a regulatory molecule. It is involved in epigenetic modification. The greatest effect of 3-HB in flax is related to DNA methylation/demethylation rather than histone acetylation/deacetylation, but it is possible that both of these processes are related to the effects of 3-HB in plants. Literature data indicate that HDAC inhibition by beta-hydroxybutyrate in animals correlates with global changes in the transcription of many genes in animal cells. In the case of plants, it seems that genes involved in the phenylpropanoid pathway may be one of the recipients of epigenetic changes induced by 3-HB [155].

## 10. Conclusions

In animal and bacterial cells 3-hydroxybutyrate is an important metabolite with mainly energetic functions. Another important function of this metabolite is the supply of acetoacetyl-CoA and acetyl-CoA for the synthesis of other important metabolites. It should be emphasized that the role of 3-hydroxybutyrate does not end with its metabolic function, but it is a molecule that exhibits a number of signaling and regulatory properties. 3-HB can perform regulatory functions directly by inhibiting HDAC and binding to cell surface receptors and indirectly by altering the levels of other regulatory metabolites. The multitude of functions of 3-HB make it a compound that enjoys interest in many aspects of human life, including in medicine, as a therapeutic compound in diseases such as those of the circulatory and nervous systems or cancer, in dietetics, as the basis of a ketogenic diet to fight, among other diseases, epilepsy and obesity, and in the biodegradable plastics industry, as a polyhydroxybutyrate monomer. In the case of plants, we have limited data, but it seems that for these organisms as well, it is an important molecule that can exhibit regulatory properties, mainly by altering DNA methylation levels.

## Data Availability

Not applicable.
